# Frailty Index as a Predictor of Mortality in Middle-Aged and Older People: A Prospective Analysis of Chilean Adults

**DOI:** 10.3390/ijerph20021195

**Published:** 2023-01-10

**Authors:** Felipe Diaz-Toro, Gabriela Nazar, Claudia Troncoso, Yeny Concha-Cisternas, Ana Maria Leiva-Ordoñez, Maria Adela Martinez-Sanguinetti, Solange Parra-Soto, Nicole Lasserre-Laso, Igor Cigarroa, Lorena Mardones, Jaime Vásquez-Gómez, Fanny Petermann-Rocha, Ximena Diaz-Martinez, Carlos Celis-Morales

**Affiliations:** 1 Department of Epidemiology, Mailman School of Public Health, Columbia University, New York, NY 10032, USA; 2 Facultad de Enfermeria, Universidad Andres Bello, Santiago 7550196, Chile; 3 Departamento de Psicología y Centro de Vida Saludable, Universidad de Concepción, Concepción 4030000, Chile; 4 Centro de Investigación en Educación y Desarrollo (CIEDE-UCSC), Departamento de Salud Pública, Facultad de Medicina, Universidad Católica de la Santísima Concepción, Concepción 4070129, Chile; 5 Escuela de Kinesiología, Facultad de Salud, Universidad Santo Tomás, Talca 8370003, Chile; 6 Pedagogía en Educación Física, Facultad de Educación, Universidad Autónoma de Chile, Talca 7500912, Chile; 7 Instituto de Anatomía, Histología y Patología, Facultad de Medicina, Universidad Austral de Chile, Valdivia 5110566, Chile; 8 Instituto de Farmacia, Facultad de Ciencias, Universidad Austral de Chile, Valdivia 5110566, Chile; 9 BHF Glasgow Cardiovascular Research Centre, School of Cardiovascular and Metabolic Health, University of Glasgow, Glasgow G12 8QQ, UK; 10 Escuela de Nutrición y Dietética, Facultad de Salud, Universidad Santo Tomás, Los Ángeles 4440000, Chile; 11 Escuela de Kinesiología, Facultad de Salud, Universidad Santo Tomás, Los Ángeles 4440000, Chile; 12 Laboratorio de Ciencias Biomédicas, Facultad de Medicina, Universidad Católica de la Santísima Concepción, Concepción 4090541, Chile; 13 Centro de Investigación de Estudios Avanzados del Maule, Universidad Católica del Maule, Talca 3460000, Chile; 14 Laboratorio de Rendimiento Humano, Education, Physical Activity and Health Research Unit, Universidad Católica del Maule, Talca 3466706, Chile; 15 Centro de Investigación Biomédica, Facultad de Medicina, Universidad Diego Portales, Santiago 8370068, Chile; 16 Grupo de Investigación Calidad de Vida, Universidad del Biobío, Chillán 4300818, Chile

**Keywords:** frailty, mortality, middle-aged, Chile

## Abstract

We aimed to investigate the association between frailty status and all-cause mortality in middle-aged and older people. We included 2661 individuals aged ≥ 35 from the Chilean National Health Survey 2009–2010. Mortality was determined through linkage with the Chilean Civil Registry and Identification. A 36-item frailty index (FI) was used to assess the frailty status. Associations between frailty status and all-cause mortality were assessed using Kaplan–Meier and Cox proportional hazard models adjusted for sociodemographic and lifestyle factors. A non-linear association was investigated using penalized cubic splines fitted in the Cox models. During an 8.9 median follow-up (interquartile range of 8.6–9.0), 308 individuals died (11.5%). Lower survival rates were observed in frail individuals compared to pre-frail and robust people (log-rank < 0.001). Compared with robust individuals, frail people had a higher mortality risk (HR: 2.35 [95% CI: 1.57 to 3.51]). Frail middle-aged individuals had a higher risk of dying independently of major risk factors.

## 1. Introduction

Frailty is a clinical-stage of increased vulnerability to developing adverse health outcomes, including falls, delirium, disability, cognitive decline, and mortality [1,2]. Its multifactorial etiology involves disorders of multiple interconnected physiological systems [3]. 

Two of the most utilized instruments to assess frailty are the frailty index (FI) [4] and the frailty phenotype (FP) developed by Fried et al. in 2001 [5]. The FI states that frailty is caused by the accumulation of health deficits during the life course and that the more deficits a person has, the more likely this person is to be frail [4]. The FI is calculated as a ratio of the number of deficits present to the number of total deficits, and can be utilized as a continuous or categorical variable [6]. Symptoms, signs, diseases, disabilities, laboratory, and from different domains (functional, cognitive, and social characteristics) are the types of deficits that can be included in the FI [4]. On the other hand, the FP identifies specific parameters that translate into a clinically relevant reduced physiological function. One remarkable weakness of the FP is that cognitive impairment—associated with functional decline and disability—is not included as a component of the FP.

The FI has been described as a well-known predictor of all-cause mortality in previous studies using different populations, independent of other major risk factors, such as age, education level, tobacco smoking, alcohol intake, among others [7,8,9,10]. Yet, its association with mortality in middle-aged adults has been less investigated; the results available suggest that younger people may have stronger associations [7,11]. However, it is unknown to what extent these findings apply to the Chilean population. 

In Chile, a cohort study (ALEXANDROS) [12], during 15 years of follow-up, reported a higher risk of death in frail individuals (hazard ratio: 1.45 [95%CI 1.04–1.90]) compared to robust people. Nonetheless, this study used the frailty phenotype instead of the FI, and the population studied were adults aged 60 years and above. To date, there is no evidence regarding the Chilean population on whether frailty is associated with a higher risk of mortality in middle-aged adults. Considering the aforementioned gaps, we aimed to investigate the association between frailty status, using a FI, and all-cause mortality in middle-aged and older Chilean adults.

## 2. Material and Methods 

### 2.1. Study Design 

This longitudinal study used data from the Chilean National Health Survey (CNHS) conducted between 2009 and 2010 [13]. The CNHS 2009–2010 is one of Chile’s largest, nationally representative population-based surveys of health conditions, lifestyles, health risk factors, and morbidity, in a stratified multistage probability sample of 5416 participants. For the current study, we included 2661 individuals aged ≥ 35 years and older with complete data for all the variables. Of the 5416 surveyed people, 1732 of them were younger than 35 years old. The other 1023 individuals ≥ 35 years old did not have complete data to construct the frailty index. Even if these participants were not included due to the aforementioned reasons, no differences in terms of sex, age, level of education and comorbidities were found in those who did not enter the study. All participants provided written consent before participation. The CNHS 2009–2010 was funded by Chilean Ministry of Health, and approved by the Ethics Research Committee of School of Medicine at the Pontificia Universidad Católica de Chile (No. 16–019).

### 2.2. Assessment of the FI 

A 36-item FI was developed based on self-reported data following the standard procedures described by Searle et al. [14]. Briefly, to be considered a deficit, a variable must satisfy the following criteria: (i) their prevalence should increase with age; (ii) be associated with health status; (iii) not saturate too early or have a very low prevalence [14]. All the variables and cut-off points included in the 36-item FI are available in the Supplementary Material. Briefly, the variables included—taken from the CNHS questionaries—are self-reported cognition (concentration and capacity of learning new skills); chronic conditions, such as acute myocardial infarction, angina, stroke, peripheral venous disease, cataracts, glaucoma, high blood pressure, diabetes, high cholesterol, chronic bronchitis/asthma, arthritis, knee osteoarthritis, hip osteoarthritis, gallbladder cancer, gastric cancer, and colon cancer; functional limitations (seven items related to difficulty performing activities of daily living); self-report of mental health (feeling down, depressed, or hopeless, suspected depression, trouble sleeping, and anxiety), self-report of health and status (three questions related to self-rated health, and perception of self-health), physical activity (assessed by the Global Physical Activity Questionnaire), anthropometry (body mass index, as measured by trained nurses), and number of falls in the last year.

All deficits were scored between 0 and 1, where 0 indicates the absence of the deficit, while 1 indicates the presence of the deficit. In addition, when an intermediate category was identified, this was categorized as 0.5.

A final frailty score was calculated for each participant by dividing the sum of the health deficit scores by the total number of health deficits assessed. Descriptive statistics using the FI score as a continuous variable were calculated for the total population. From the continuous score, three categories were created following the cut-off points proposed by Rockwood et al. [15]. These were as follows: (i) <0.12 points, robust; (ii) >0.12 to 0.24 points, pre-frail; and (iii) >0.24 frail. More information about all the variables and cut-off points included is available in Supplementary Material Table S1.

### 2.3. All-Cause Mortality 

Long-term follow-up for all-cause mortality data, including dates of death, were obtained from linkages of the CNHS to the Chilean Civil Registry and Identification. Mortality data were available until 26 December 2018. Therefore, mortality was based on this date or the date of death. 

### 2.4. Covariates

Self-reported data for sociodemographic characteristics, including age, sex, years of education, place of residency, smoking status, and alcohol consumption, were collected from all participants using questionnaires previously validated for the CNHS 2009–10. The following categories were derived for the sociodemographic variables: age (<60 or ≥60), sex (men or women), years of education (≤8 years, 9–12 years, or >12 years), place of residence (urban or rural), and smoking status (never, previous, or current). Alcohol consumption was derived using the Alcohol Use Disorders Identification Test (AUDIT) [16], and categorized as low, moderate, high, and dependence risk.

### 2.5. Statistical Analyses 

Baseline characteristics were presented according to the frailty status (robust, pre-frail, and frail) as mean and standard deviation (SD) for continuous variables, and percentages with their 95% confidence intervals (CI) for categorical variables. 

Crude Kaplan–Meier curves were constructed to estimate the 9-year survival for categories of the FI for the general population and stratified by sex and age group.

A test of proportional hazards assumption was conducted (*p* = 0.374), and Cox proportional regression for the overall population and stratified by sex and age group were performed to investigate the association between frailty status and mortality. Results are reported as hazard ratio (HR) with their respective 95% CI. For the analyses, robust people were used as the reference group. 

Three models with an incremental number of covariates were conducted; model 1 was unadjusted, model 2 was adjusted for age, sex, years of education, and place of residence, and model 3 was additionally adjusted for smoking status and AUDIT score. 

A non-linear association between the continuous FI and all-cause mortality was also investigated using penalized cubic splines fitted in Cox proportional hazard models. The penalized spline is a variation of the basis spline, which is less sensitive to known numbers and placements than restricted cubic splines [17]. For this spline, values were truncated to less than 1% of the values for FI. After truncation, the minimum value of the FI was 0.1. In addition, the mean value of the FI was used as the reference group. All statistical analyses were conducted using STATA V17 software (StataCorp; College Station, TX, USA) and R 3.6.1 (using the packages ‘survival’, and ‘spline’). A *p*-value below 0.05 was considered statistically significant.

## 3. Results

**Figure 1:**
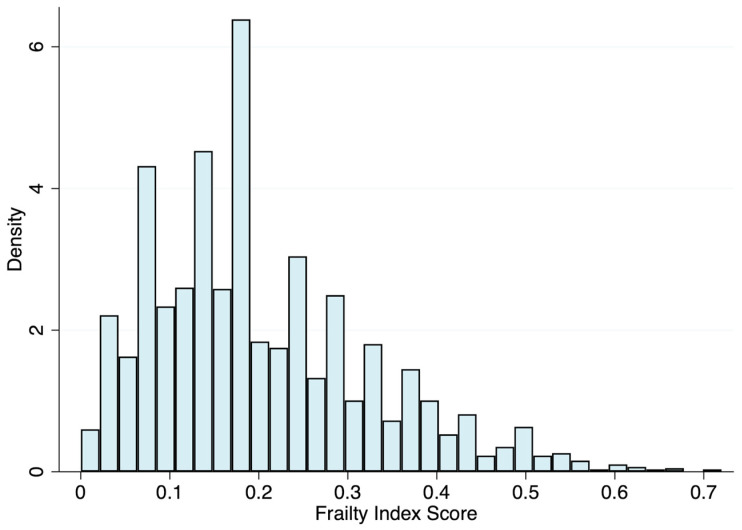
Distribution of the 36-item frailty index.

Figure 1 shows the distribution of the data, which skewed to the right. From the continuous scale, the mean FI score was 0.20 (SD = 0.12), with a median of 0.18, ranging from 0 to 0.72, and a 99% upper limit of 0.56. The characteristics of the study population by frailty status are shown in Table 1. Of the 2661 participants included in this analysis, 39.2% and 29.3% were classified as pre-frail and frail, respectively. Briefly, compared with robust individuals, pre-frail and frail people were older, more likely to be women, tended to have ≤8 years of education, and were more likely to have a higher alcohol intake. 

**Figure 2:**
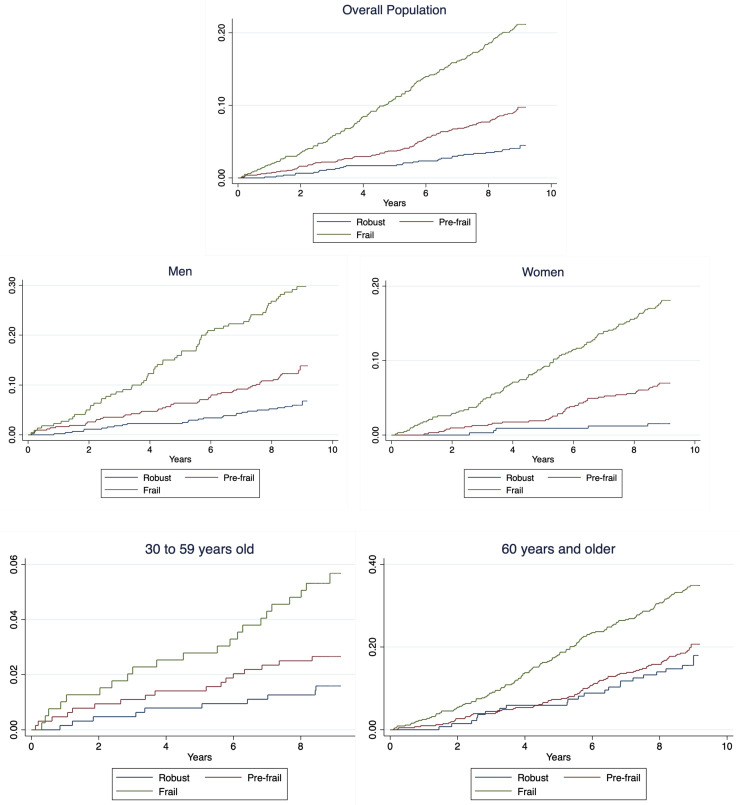
Kaplan–Meier failure estimates for general population and stratified by sex.

During an 8.9-year median follow-up (interquartile range: 8.6–9.0), 308 individuals died (11.5%). Kaplan–Meier failure estimates by frailty status for the general population and stratified by sex and age group are shown in Figure 2. Briefly, for all the groups, lower survival rates in frail and pre-frail individuals compared to robust were observed (log-rank < 0.001).

Associations between the frailty status and all-cause mortality, stratified by sex and age group, are shown in Table 2. In the unadjusted model, and compared with robust participants, frail individuals had a 5.51-times higher risk of death (HR _model 1:_ 5.51 [95%CI 3.78 to 8.04]). The association was attenuated after adjusting for the covariates included in models 2 (sociodemographic) and 3 (lifestyle); however, it remained statistically significant (HR _model 3:_ 2.35 [95%CI 1.57 to 3.51]). The mortality risk in pre-frail individuals was 2.34-times higher than in robust participants for the unadjusted model. However, this association was fully attenuated after further adjustment (HR:1.28 [95%CI 0.85 to 1.92]). In sex-stratified models, the risk of dying was higher in frail women than frail men (HR _women model 3_: 3.59 [95% CI: 1.44 to 8.93] vs. HR_men model 3_: 2.06 (95% CI: 1.28 to 3.30). Furthermore, for people <60 and ≥60, the risk of dying was higher for frail than robust individuals after adjustment for all confounders (HR_model 3_: 3.54 [95% CI: 1.58 to 7.90]; 2.62 [95% CI: 1.65 to 4.14], respectively). 

**Figure 3:**
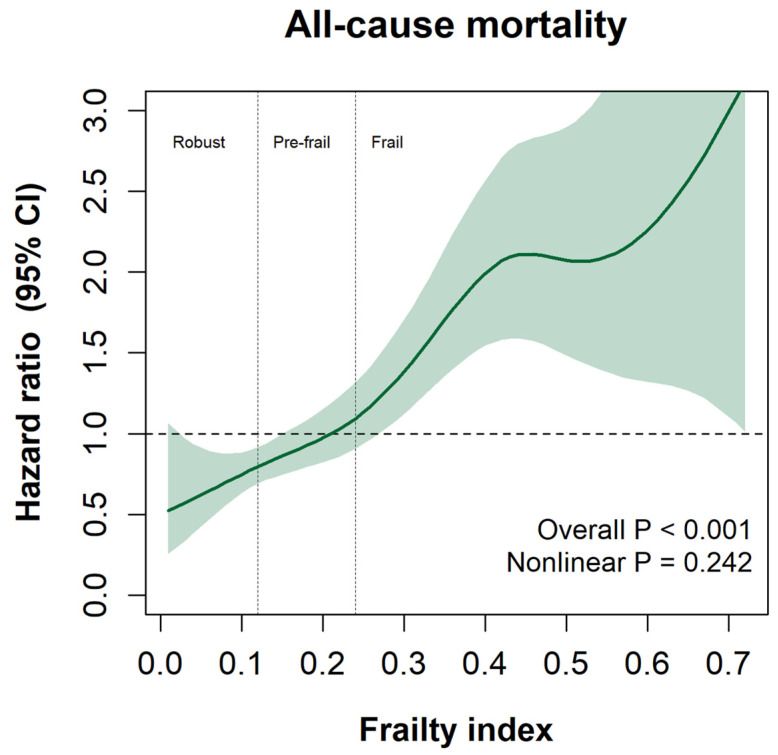
Spline graph is presented as HR and their 95% CI. Frailty index is presented as a continuous variable. All analyses were adjusted for age, sex, zone of residence, smoking status, and AUDIT score.

The non-linear association between the continuous FI score and all-cause mortality is shown in Figure 3. Overall, a higher FI score was associated with a higher risk of mortality (overall *p* < 0.001). No evidence of non-linearity was observed in the spline. 

## 4. Discussion

Using a 36-item FI, frail middle-aged Chileans had a higher risk of dying than robust people, even after adjusting for a wide range of confounder variables. To our knowledge, this is the first study to explore the association between frailty status and mortality risk using the middle-aged and older Chilean population. In this study, the observed risk was higher in women and in middle-aged people (<60 years), which is consistent with the literature that reports higher mortality rates in frail younger populations. Our results suggest that earlier detection of pre-frail and frail individuals may represent an opportunity to introduce effective management strategies to improve outcomes—including reducing mortality—in middle-aged and older people.

The association between frailty status and all-cause mortality has been extensively studied in older populations [18]. These studies have been conclusive, stating that frail people aged 65 and older have a higher mortality risk than the general population [18]. Yet, even if frailty is usually associated with older people (because it is a condition that comprises age-related changes due to the lifelong accumulation and exposition of cellular and molecular damage [19,20]) previous studies have highlighted that frailty is a process that starts earlier and may also be present in middle-aged individuals [21,22].

In this line, few studies have shown that a higher FI is associated with a greater risk of all-cause mortality in individuals younger than 60 years. For instance, Junning Fan et al. [23], after following 512,273 Chinese participants aged 30 and older for 10.8 years, identified that the association between a 28-item FI and all-cause mortality, was stronger in middle-aged individuals (<50 years) than in older participants (HR_<50_: 1. 95 [95% CI: 1.87 to 2.03]; HR _50–64_: 1.80 [95% CI: 1.76 to 1.83]; and HR_>65_: 1.56 [95% CI: 1.53 to 1.59]) [23]. Similarly, Jiang et al. [24], when following 1477 individuals older than 29 years old, reported that, after adjustment for covariates, a 42-item FI was independently associated with increased risk for all-cause mortality in women and men aged < 65 years (HR 1.11 [95% CI: 1.07 to 1.17]; 1.05 [95% CI: 1.01 to 1.10], respectively). On the other hand, for women and men aged ≥ 65 years, the HRs were HR_men_ 1.07 [95%CI 1.04–1.10]; and HR_women_ 1.03 [0.99 to 1.07]. Likewise, Williams et al. [25], when using a 49-item FI, showed that the HRs for mortality were stronger in younger participants than in older participants (HR_<50_ 1.87 [95% CI: 1.74 to 2.00], and HR_>65_ 1.59 [95% CI: 1.54 to 1.64]) after adjustment for sociodemographic characteristics [25]. Explanations for these findings might respond to an accelerated and well-documented [23] aging process in younger individuals, but could also be due to the presence of more comorbidities or chronic conditions related to unhealthy lifestyle behaviors. We cannot exclude the survivors’ effect in older people, which can result from better medical treatments that increase the expectancy of life and quality of life. 

In terms of sex differences, our study showed that the risk of all-cause mortality was higher in frail women than frail men. These findings agree with those published by Jiang et al. [24], where frail women were at higher risk of mortality than men (HR_men_ 1.08 [95% CI: 1.06 to 1.11]: HR_women_ 1.04 [95% CI: 1.01 to 1.07]). Conversely, our results differ from those published by Gordon et al. [26] and Williams et al. [25], in which the risk of mortality was higher in frail men than women, even when a higher prevalence of frail status in women was reported.

The higher mortality risk of participants younger than 60 compared with older individuals observed in this report might be partially explained by Chilean disease burden and life expectancy. The FI is characterized by the accumulation of deficits [15]. These deficits generally increase with age; however, over the last years, the prevalence of non-communicable diseases, unhealthy behaviors and habits, as well as other disabilities, have increased in people younger than 60 years old [27], which may explain the higher risk of mortality compared with older individuals observed in this report. Moreover, in Chile, women tend to live longer than men (82.2 vs. 76 years) [28], but with a higher burden of disabilities—the male–female health–survival paradox [29]. Therefore, a greater deficit in women than in men could have triggered a higher mortality rate in this group. 

### Strengths and Limitations

This is the first study investigating the association between frailty and all-cause mortality in middle-aged and older Chileans. Moreover, we used representative data from the Chilean population and included an extensive number of people from different geographic zones of Chile. Frailty status was assessed using a FI that included questionaries and scales validated in the country, covering a wide range of health domains, and following standard procedures previously described, ensuring the validity and replicability of these findings. 

However, this study has limitations. First, some variables, such as comorbidities, functional limitations, and mental health status, were self-reported, which might be subject to recall bias and underestimate the prevalence of frailty status. Second, frailty status and confounders were assessed only at baseline; therefore, bias for adjusting for potential mediators may be present, and we could not account for potential changes in those variables’ status over the follow-up. Third, the observational nature of the data does not allow infer causality from the results. Finally, even when we adjust for important confounders, the effect of unmeasured confounders, such as a comprehensive cognitive assessment and muscular measurements, cannot be ruled out.

## 5. Conclusions

Using the CHNS 2009–2010, frailty was associated with all-cause mortality even after adjustment for confounders. Moreover, the associations appear to be stronger in younger rather than older participants, and in women rather than men. This is the first paper investigating the relationship between a frailty index and mortality in both middle-aged and older people in Chile and in Latin America. 

Early detection of frailty, using multiple variables, could be useful as an indicator to consider in developing public health policies focused on health promotion in middle-aged Chilean people. Further research is needed to explore the association between the FI and specific causes of mortality, and to examine whether interventions may impact the development of future outcomes. 

## Figures and Tables

**Table 1:** Baseline characteristics of the study participants by frailty status (N = 2661).

	Total	Robust	Pre-Frail	Frail	*p*-Value
		(N = 769)	(N = 1048)	(N = 844)	
		% (95%CI)	% (95%CI)	% (95%CI)	
All		31.5 (28.2–34.8)	39.2 (35.8–42.5)	29.3 (26.5–32.3)	
Age (mean, SD)	55.4 (13.6)	49.1 (11.1)	55.9 (13.2)	60.9 (13.8)	<0.001
Age groups (%)					
35–44	26.1 (24.5–27.9)	53.6 (47.2–60.0)	34.5 (29.3–40.1)	19.3 (14.9–24.7)	<0.001
45–54	25.5 (23.9–27.2)	25.4 (20.9–30.6)	26.7 (22.1–31.9)	24.5 (19.7–29.9)	
55–64	21.3 (19.8–22.9)	13.5 (10.1–17.9)	18.6 (15.2–22.7)	24.4 (20.2–29.2)	
65–74	15.6 (14.3–17.1)	5.3 (3.1–8.8)	13.7 (10.6–17.7)	16.8 (13.3–21.1)	
75 and older	11.3 (10.2–12.5)	2.1 (0.9–4.1)	6.3 (4.3–9.1)	14.9 (11.3–19.2)	
Sex (%)					
Men	40.7 (38.9–42.6)	63.1 (56.8–68.9)	48.2 (42.7–53.6)	30.2 (24.9–36.1)	<0.001
Women	59.3 (57.3–61.1)	36.9 (31–43.2)	51.8 (46.4–57.2)	69.8 (63.9–75.1)	
Years of education (%)					
≤8 years	36.4 (34.6–38.2)	28.3 (22.8–34.5)	36.5 (31.8–41.5)	54.6 (48.9–60.3)	0.003
9–12 years	47.9 (46–49.8)	43.7 (37.4–50.3)	38.4 (33.4–43.7)	36.1 (30.6–42.1)	
>12 years	15.7 (14.3–17.1)	27.9 (21.8–35.1)	25 (19.9–30.9)	9.2 (6.6–12.6)	
Place of residence (%)					
Urban	83.9 (82.5–85.3)	89.5 (86.3–92.1)	86.8 (83.8–89.2)	83.7 (79.6–87.1)	0.872
Rural	16.1 (14.7–17.5)	10.5 (7.9–13.7)	13.3 (10.8–16.2)	16.3 (12.9–20.4)	
Smoking (%)					
Never	28.2 (26.6–30.1)	40.2 (33.9–46.9)	33.2 (28.7–38.1)	36.5 (31.5–41.9)	0.638
Previous	42.1 (40.1–43.9)	22.5 (17.8–27.8)	33.2 (28.2–38.7)	28.6 (24.1–33.6)	
Current	29.7 (28–31.5)	37.2 (30.8–44.1)	33.5 (28.4–39)	34.8 (29.3–40.8)	
AUDIT Score (%)					
Low risk	92.5 (91.4–93.4)	90.5 (86.6–93.5)	91.5 (88.3–93.9)	93 (89.5–95.4)	0.761
Moderate risk	5.9 (5.1–6.8)	7.9 (5.2–11.7)	6.8 (4.7–9.7)	4.1 (2.6–6.5)	
High risk	1.0 (0.6–1.5)	1.1 (0.3–3.3)	1.4 (0.5–3.5)	1.2 (0.2–5.9)	
Dependence	0.6 (0.4–1.0)	0.4 (0.1–1.5)	0.2 (0.04–0.8)	1.6 (0.7–3.5)	
Mortality, yes (%)	11.5 (10.3–12.7)	2.6 (1.5–4.5)	7.1 (1.5–9.7)	15.2 (12.1–18.9)	<0.001

Continuous variables are expressed as mean and standard deviation (SD). Categorical variables are expressed as percentages with their 95% confidence intervals (95%CI).

**Table 2:** Association between frailty status and risk of all-cause mortality by sex and age categories.

	Model 1	Model 2	Model 3	
	HR (95% CI)	HR (95% CI)	HR (95% CI)	*p*-Interaction
Overall (N = 2661)				-
Robust (Ref.)	1.00	1.00	1.00	
Pre-frail	2.34 (1.57–3.49)	1.28 (0.85–3.51)	1.28 (0.85–1.92)	
Frail	5.51 (3.78–8.04)	2.35 (1.58–3.51)	2.35 (1.57–3.51)	
Men (N = 1085)				*p* < 0.001
Robust (Ref.)	1.00	1.00	1.00	
Pre-frail	2.23 (1.41–3.52)	1.24 (0.78–1.99)	1.25 (0.78–1.99)	
Frail	5.55 (3.54–8.69)	2.05 (1.28–3.29)	2.06 (1.28–3.30)	
Women (N = 1576)				
Robust (Ref.)	1.00	1.00	1.00	
Pre-frail	4.62 (1.83–11.6)	1.88 (0.74–4.79)	1.86 (0.73–4.73)	
Frail	12.84 (5.24–31.5)	3.67 (1.47–9.10)	3.59 (1.44–8.93)	
Age 35 to 59 years (N = 1668)				*p* < 0.001
Robust (Ref.)	1.00	1.00	1.00	
Pre-frail	1.69 (0.77–3.70)	1.76 (0.79–3.88)	1.72 (0.78–3.80)	
Frail	3.60 (1.70–7.60)	3.84 (1.73–8.54)	3.54 (1.58–7.90)	
Age ≥ 60 years (N = 993)				
Robust (Ref.)	1.00	1.00	1.00	
Pre-frail	1.26 (0.79–2.02)	1.31 (0.81–2.11)	1.31 (0.81–2.12)	
Frail	2.41 (1.54–3.78)	2.61 (1.65–4.12)	2.62 (1.65–4.14)	

Model 1—unadjusted. Model 2—adjusted for years of education and place of residence. Model 3—as per model 2 and also account for smoking status and AUDIT score. Analyses are presented as HR and their 95% CI.

## Data Availability

These data were derived from the following resources available in the public domain: website of the Epidemiology Department of the Ministry of Health, Chile: http//epi.minsal.cl/encuesta-ens/ (accessed on 1 October 2022).

## Supplementary Materials

The following supporting information can be downloaded at: https://www.mdpi.com/article/10.3390/ijerph20021195/s1, Table S1. Type of deficits included for the frailty index.
