# Supplementary material for: Frailty Index as a Predictor of Mortality in Middle-Aged and Older People: A Prospective Analysis of Chilean Adults
Source: Int J Environ Res Public Health. 2023 Jan 10;20(2):1195. doi: 10.3390/ijerph20021195 (PMC9859421; doi:10.3390/ijerph20021195)
Supplement: Supplementary file 1 [file ijerph-20-01195-s001.zip › ijerph-2026767-supplementary.pdf]

Table S1. Type of deficits included for the frailty index.

Type of Deficit	Scoring	Prevalence of the Deficit
COGNITION		
Concentration	None = 0, mild/moderate = 0.5, severe/extreme = 1	None = 43.3%, mild/moderate = 47.4%, severe/extreme = 9.3%
Capacity of learning new skills	None = 0, mild/moderate = 0.5, severe/extreme = 1	None = 58.7%, mild/moderate = 33.8%, severe/extreme = 7.6%
COMORBIDITIES		
Acute myocardial infarction	Yes = 1, No = 0	Yes = 24.2%
Angina	Yes = 1, No = 0	Yes = 15.4%
Stroke	Yes = 1, No = 0	Yes = 17.1%
Peripheral venous disease	Yes = 1, No = 0	Yes = 8.3%
Cataracts	Yes = 1, No = 0	Yes = 8.5%
Glaucoma	Yes = 1, No = 0	Yes = 2.9%
High blood pressure	Yes = 1, No = 0	Yes = 42.5%
Diabetes	Yes = 1, No = 0	Yes = 10.3%
High cholesterol	Yes = 1, No = 0	Yes = 27.6%
Chronic bronchitis/asthma	Yes = 1, No = 0	Yes = 11.1%
Arthritis	Yes = 1, No = 0	Yes = 4.4%
Knee osteoarthritis	Yes = 1, No = 0	Yes = 6.3%
Hip osteoarthritis	Yes = 1, No = 0	Yes = 4.1%
Gastric cancer	Yes = 1, No = 0	Yes = 4.1%
Colon cancer	Yes = 1, No = 0	Yes = 13.9%
Bladder cancer	Yes = 1, No = 0	Yes = 6.7%
DIFFICULTY FOR		
Walking or climbing steps	None = 0, mild/moderate = 0.5, severe/extreme = 1	None = 65.8%, mild/moderate = 26.6%, severe/extreme = 7.6%
Dressing and bathing	None = 0, mild/moderate = 0.5, severe/extreme = 1	None = 93.9%, mild/moderate = 3.6%, severe/extreme = 2.5%
Personal aspects (shaving, brushing, etc.)	None = 0, mild/moderate = 0.5, severe/extreme = 1	None = 94.8%, mild/moderate = 3.3%, severe/extreme = 1.9%
Going out of the house	None = 0, mild/moderate = 0.5, severe/extreme = 1	None = 82.1%, mild/moderate = 13.9%, severe/extreme = 4%
Community activities	None = 0, mild/moderate = 0.5, severe/extreme = 1	None = 69.9%, mild/moderate = 17.7%, severe/extreme = 12.4%
House chores	None = 0, mild/moderate = 0.5, severe/extreme = 1	None = 57.3%, mild/moderate = 27.5%, severe/extreme = 15.2%
Intense activities	None = 0, mild/moderate = 0.5, severe/extreme = 1	None = 46.5% mild/moderate = 14.1%, severe/extreme = 39.4%
Trouble solving problems	Not at all = 0, Moderate = 0.5, Very = 1	Not at all = 83.4%, Moderate = 10.2%, Very = 6.4%
MENTAL HEALTH		
Feeling down, depressed, or hopeless	Not at all = 0, Moderate = 0.5, Very = 1	Not at all = 54.8%, Moderate = 29.8%, Very = 15.4%

Suspected depression	Yes = 1, No = 0	Yes = 15.9%
Trouble sleeping	Yes = 1, No = 0	Yes = 35.1%
Anxiety	Yes = 1, No = 0	Yes = 38.6%
SELF-REPORT OF HEALTH AND STATUS		
Self-rated heath	Very good/good = 0, fair = 0.5, poor/very poor = 1	Very good/good = 49.2% fair = 42.3%, poor/very poor = 8.5%
Perception of personal energy	Very good/good = 0, fair = 0.5, poor/very poor = 1	Very good/good = 59.2%, fair = 37.6%, poor/very poor = 3.2%
Self-rated pain	Never = 0, rarely/sometimes = 0.5, usually = 1	Never = 27.9%, rarely/sometimes = 55.3% usually = 16.8%
ANTHROPOMETRY AND OTHERS		
Body mass index	Underweight/obese = 1, overweight = 0.5, healthy = 0	Underweight/obese = 30.7%, overweight = 40.1%, healthy = 29.2%
Falls in last year	Yes = 1, No = 0	Yes = 28.6%
Physical activity	80th percentile = 0, other = 1	Other = 25.5%
